# Identification of Ferroptosis‐related molecular model and immune subtypes of hepatocellular carcinoma for individual therapy

**DOI:** 10.1002/cam4.5032

**Published:** 2022-07-16

**Authors:** Shichao Long, Yuqiao Chen, Ya Wang, Yuanbing Yao, Shuai Xiao, Kai Fu

**Affiliations:** ^1^ Department of General Surgery, Institute of Molecular Precision Medicine and Hunan Key Laboratory of Molecular Precision Medicine Xiangya Hospital, Central South University Changsha Hunan China; ^2^ The First Affiliated Hospital, Institute of Oncology, Hengyang Medical School, University of South China Hengyang Hunan China; ^3^ Center for Medical Genetics & Hunan Key Laboratory of Medical Genetics, School of Life Sciences Central South University Changsha Hunan China; ^4^ Hunan Key Laboratory of Animal Models for Human Diseases Central South University Changsha Hunan China; ^5^ National Clinical Research Center for Geriatric Disorders Changsha Hunan China; ^6^ Hunan Key Laboratory of Aging Biology Xiangya Hospital, Central South University Changsha Hunan China

**Keywords:** ferroptosis, hepatocellular carcinoma, molecular subtype

## Abstract

**Background:**

Excessive iron accumulation and lipid peroxidation are primary characteristics of ferroptosis in hepatocellular carcinoma (HCC). Ferroptosis inducer combined with immunotherapy has become a new hope for HCC patients. Therefore, the construction and validation of subtype‐specific sensitivity to ferroptosis inducer will be helpful for hierarchical management and precise individual therapy.

**Methods:**

RNA‐seq transcriptome and clinical data of HCC patients were extracted from International Cancer Genome Consortium (ICGC) dataset and The Cancer Genome Atlas (TCGA) dataset. Consistency matrix and data clustering of the both cohorts were constructed by ‘ConsensusClusterPlus’ package. Single‐sample gene set enrichment analysis (ssGSEA) analysis was performed to evaluate immune infiltration. Cox analysis was utilized to construct a ferroptosis phenotype‐related prognostic model (FRPM) in HCC. The predictive efficiency of the constructed FRPM was evaluated through Kaplan Meier (K‐M) survival analyses and Receiver Operating Characteristic (ROC) curves. The expression levels of candidate genes were detected and validated by Real‐Time PCR between liver cancer tissues and adjacent non‐tumor liver tissues.

**Results:**

45 differentially expressed ferroptosis‐related genes (FRGs) were identified between HCC tissues and non‐tumor liver tissues. Furthermore, four ferroptosis‐associated clusters (FACs) of HCC were established via consensus clustering. Subsequently, we established a FRPM, consisting of four prognostic genes (*SLC7A11*, *SLC1A5*, *GCLM* and *SAT1*), to evaluate the survival of HCC patients, based on which, patients were divided into high‐risk group and low‐risk group. The high‐risk group exhibited worse survival compared to low‐risk group (*p* < 0.0001 both in TCGA and ICGC cohorts). Patients belong to different FACs or different risk scores showed distinct clinical characteristics. Moreover, in the validation experiment, the transcriptional expression levels of the four prognostic genes were consistent with the results drew from datasets.

**Conclusion:**

We revealed a novel FRGs signature, which may provide the molecular characteristic data for effectively prognostic evaluation and potential personalized therapy for HCC patients.

## Graphical abstract

In the present analysis, we established a novel Cox proportional hazards regression model based on four ferroptosis‐related genes to evaluate the overall survival rate of patients with hepatocellular cancer. We revealed a novel FRGs signature, which may provide the molecular characteristic data for effectively prognostic evaluation and potential personalized therapy for HCC patients.

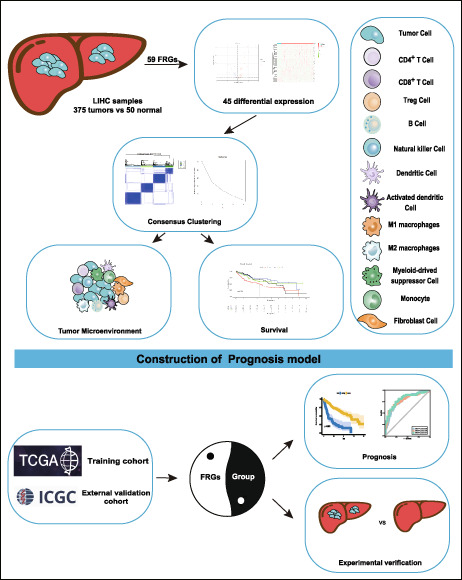

## 1 INTRODUCTION

Hepatocellular carcinoma (HCC) is the predominant subtype of primary liver cancer.^1^ HCC in worldwide is the fourth most common cause of cancer‐related death and the sixth most common in terms of incidence.^2^ Over 1 million patients are estimated to die of HCC in 2030, according to the World Health Organization's annual projections.^1^ In the United States, from 2000 to 2016, the five‐year HCC patient's survival rate is 18%.^3^ Therefore, it is of significant clinical value and social significance to find sensitive and efficient prognostic assessment methods, and precise and personalized targeted treatment strategies.

Ferroptosis, a newly noticed manner of cell death, is driven for abnormal cellular iron overload and iron‐dependent lipid peroxidation.^4^ Excessive or defective ferroptosis can contribute to tumorigenesis, epithelial‐mesenchymal transition, and various of systems diseases.^5^, ^6^ Ferroptosis can be induced through two main ways: the intrinsic or enzyme‐mediated form, as well as the external or transporter‐dependent pathway.^7^ The abnormally enhanced iron amassing, iron‐dependent reactive oxygen species and lipid peroxidation by characteristic enzymes are pivotal in occurrence of ferroptosis.^6^, ^7^ Cancer cells exhibit abnormally increased iron accumulation to enable their rapid growth compared with noncancer cells.^8^ Indeed, the HCC patients with nonalcoholic steatohepatitis‐associated cirrhosis are more frequent than non‐HCC patients.^9^ Sorafenib is an effective medicine to kill HCC cells in a ferroptosis dependent manner. Nevertheless, the therapeutic efficacy of sorafenib is significantly inhibited by ferroptosis‐related inhibitor (ferrostatin‐1, DFX, and genetic procedures).^10^, ^11^ More emerging evidence suggest that induction of ferroptosis in tumor cells may be an effective tumor therapy.^5^, ^12^, ^13^ So far, the prognostic model of varied ferroptosis‐associated tumors show an excellent prognostic efficiency.^14^, ^15^, ^16^, ^17^, ^18^, ^19^ However, prognostic value of key regulators of ferroptosis in HCC is largely unclear.

The purpose of the herein study is to establish a novel ferroptosis‐associated HCC model to select suitable patients for precision treatment. Four robust ferroptosis‐associated clusters (FACs) and four candidate genes are correlated with overall survival of HCC from TCGA dataset, which are validated in another independent cohort of Liver Cancer‐RIKEN derived from International Cancer Genome Consortium (ICGC) dataset. We then exploit the immune landscape of FACs to provide a theoretical basis for selecting patients suitable for combined immunotherapy. Our results demonstrate that each subtype has the distinct clinical features, molecular characteristics, and prognosis. Finally, the molecular characteristics of the 4 candidate genes are experimentally validated in clinical samples.

## 2 MATERIAL AND METHODS

### 2.1 Data acquisition and procession

**FIGURE 1:**
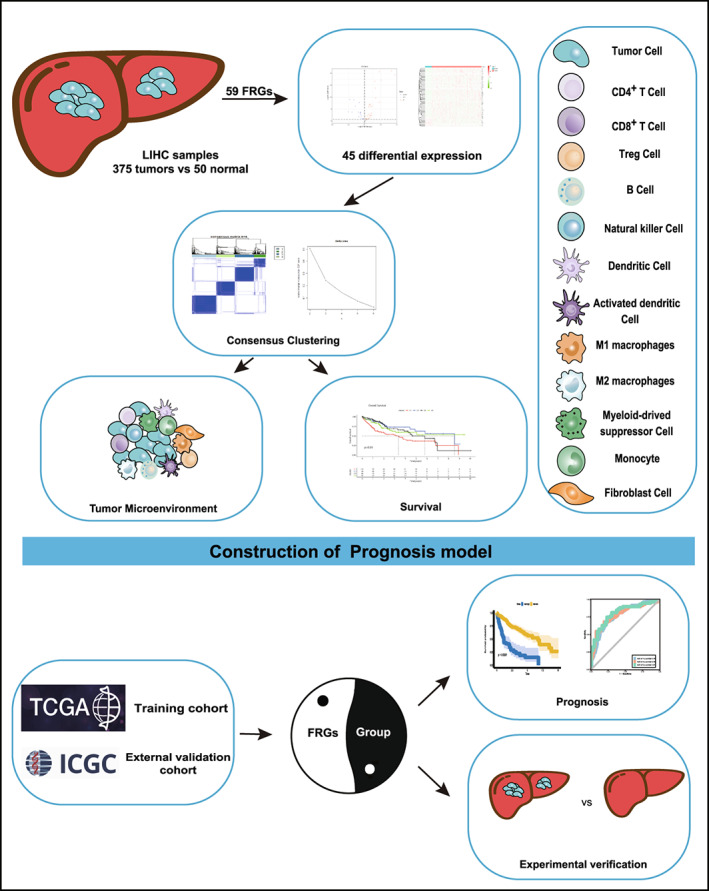
The workflow of the data collection and analysis of Ferroptosis related genes (FRGs) in LIHC.

The gene expression of RNA‐seq data (normalized count) and corresponding clinical phenotype information of HCC were extracted from LIHC of TCGA dataset (https://xenabrowser.net/datapages/) and ICGC dataset (https://dcc.icgc.org). Both the ‘DESeq2’ and the ‘limma’ packages were untilized to identify ferroptosis‐related differentially expressed genes (FDEGs) between HCC and normal tissues by R software. Next, patients were divided into four clusters based on consensus clustering analysis, which exhibit differential tumor microenvironment and survival character. To illuminate further classification, we constructed a risk model related to ferroptosis in HCC and explored mRNA level of the risk gene between liver cancer and adjacent normal tissues (Figure 1). Last, six pairs of fresh frozen HCCs and adjacent nontumor liver tissues (ANLTs) from HCC patients with liver resection between January 2018 and January 2021, from The First Affiliated Hospital of University of South China (USC), were randomly selected for independent sample validation. The metadata of the HCC patients were displayed in the Table S1. The human renunciative pathological tissues used in study were approved by the ethics committee of The First Affiliated Hospital of USC and were in accordance with the Declaration of Helsinki.

### 2.2 Consensus clustering

The false‐discovery rate (FDR) to adjust the *p* < 0.05 and set absolute log_2_‐fold change (|log_2_FC|) > 0 as cutoff criteria for FDEGs in both cohorts. Samples from TCGA or ICGC were split into different clusters based on FDEGs using the ‘ConsensusClusterPlus’ R package.

### 2.3 Construction and external validation of the risk predictive model

To screened a survival prediction signature based on FDEGs, the ‘survival’ package was employed to perform univariate Cox regression analysis using a filtering criterion of *p* < 0.05. Subsequently, a model was constructed by multivariate Cox regression analysis. Finally, we obtained the top four prognostic genes among FDEGs and therewith obtained a risk score formula: $e^{\mathrm{sum}\;\left(\text{each}\;\mathrm{gen}e^{\prime }s\;\text{expression}\times \text{corresponding coefficient}\right)}$. The optimal cutoff value was defined as the calculated risk scores of HCC samples in both cohorts using the ‘survminer’ package, and then patients in the TCGA (*n* = 368) and ICGC (*n* = 445) dataset were divided into high‐ and low‐risk group. The ‘rms’ package was used to constructed and visualized the nomogram.

### 2.4 Survival analysis

The K‐M survival analysises and relevant diagrams were carried out using the ‘survival’ and the “survminer” package. The prognostic efficiency of the model was verified by time‐dependent ROC curves. The calculated method of risk scores of Tang and Liang were extracted from their studies.^20^, ^21^

### 2.5 The evaluation of immune landscape

The single‐sample gene set enrichment analysis (ssGSEA) analysis was used to calculate 28 immune enrichment scores for each FACs.^22^ Immune microenvironment scores of samples in LIHC‐TCGA dataset derived from ESTIMATE (https://bioinformatics.mdanderson.org/estimate/) website, which include immune score (reflecting the degree of immune cell infiltration), stromal score (reflecting the degree of tumor stromal cell infiltration), tumor purity (combined tumor extracellular matrix and immune cell to infer the purity of tumor), and estimate score (reflecting tumor immune microenvironment).^23^ The z‐scale of immune checkpoint molecules in each cluster was showed in a boxplot. Genetic mutation data of samples in LIHC‐TCGA were downloaded from the cBio Cancer Genomics Portal (http://www.cbioportal.org), and then genetic mutations alterations in LIHC‐TCGA dataset were compared.

### 2.6 Real‐time PCR

The total RNA was extracted from the 6 pairs of HCCs and ANLTs using RNA Isolator Total RNA Extraction Reagent (Vazyme). The cDNA was synthesized from total RNA using NovoScript® Plus All‐in‐one 1st Strand cDNA Synthesis kit (Novoprotein). Then RT‐PCR was performed using the SYBR Green Master Mix (Yeasen) and ACTB was employed as an internal control. The primers used for RT‐PCR detection are listed in the Table S2.

### 2.7 Statistics

Statistical analysis was accomplished by R version 4.0.1. The DEGs were obtained using the one‐way ANOVA method. The Kaplan–Meier method and bilateral log‐rank test were used to calculate the gap statistics of different group. Differences between groups were analyzed using a Kruskal–Wallis test or Student's *t*‐test.

## 3 RESULTS

### 3.1 Identification of FDEGs in HCC

**FIGURE 2:**
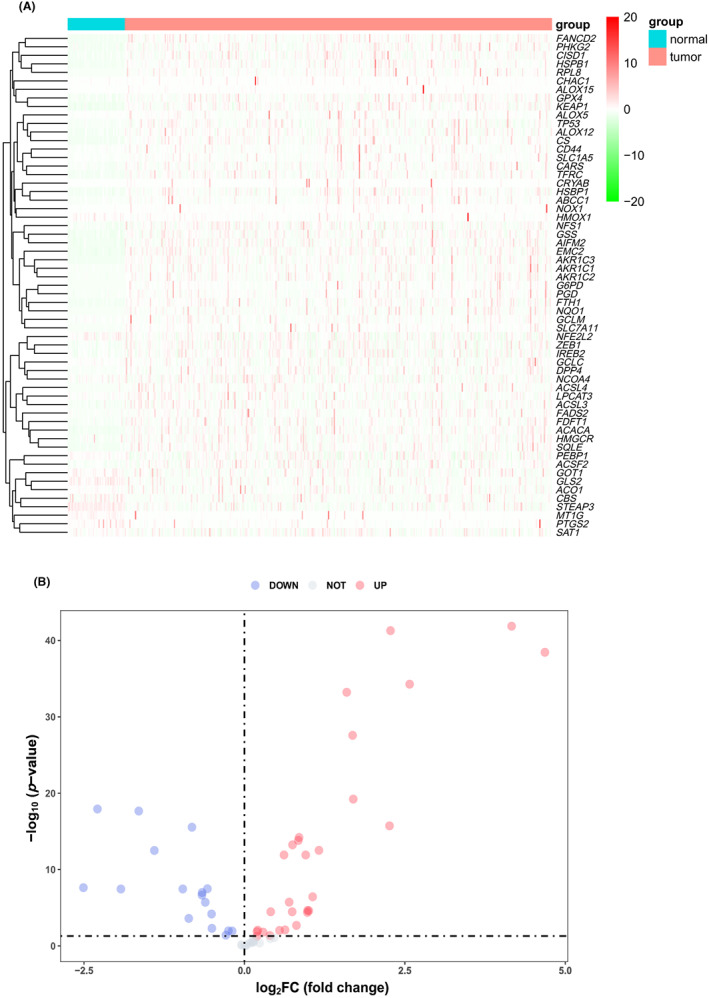
Identification of ferroptosis‐related differentially expressed genes (FDEGs) in TCGA‐LIHC patients. (A) The heatmap represents a FDEGs between 375 liver cancer and 50 adjacent non‐tumor liver tissues. (B) The volcano plot of 45 FDEGs in LIHC patients. The orange points reflect upregulated FRGs, and the blue points reflect downregulated FRGs in the plot, respectively.

It's reported that 59 FRGs in a variety of tumors were included.^14^ Moreover, we further confirmed 45 FDEGs using the condition of |log_2_FC| > 0 and FDR < 0.05 in LIHC‐TCGA samples (Figure 2A,B).

### 3.2 Classification of FDEGs clusters in LIHC

**FIGURE 3:**
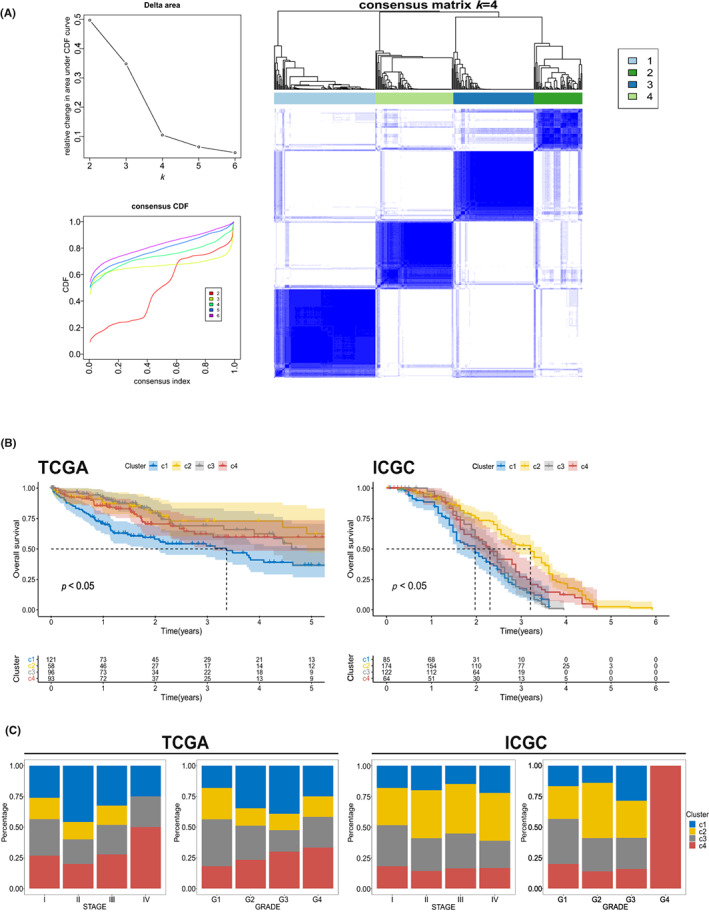
Classification of a ferroptosis‐associated clusters (FACs) of LIHC. (A) Delta area, cumulative distribution function curve and consensus clustering matrix (*k* = 4) of FRGs in TCGA cohort (*n* = 368). (B) Kaplan–Meier curves show overall survival (OS) of different clusters in the TCGA cohort and ICGC cohort (*n* = 445). (C) Distribution of c1‐c4 across LIHC stages and grades in TCGA and ICGC cohort.

Model construction based on FDEGs can be used to identify the ferroptosis status, and thus help screen suitable patients for treatment. To obtain an accurate cluster, we referred to the cumulative distribution function and function delta area. Then, we regarded specified cluster counts (*k* = 4) as stable subtype (Figure 3A), and acquired 4 FACs. The transcriptomic characteristics of these four categories of patients were shown in Figure S1. As shown in Figure 3B, cluster 1 is linked with a worse two‐year OS (*p* < 0.05, Figure 3B). Cluster distribution differs from tumor stages and grades, whereas cluster 1 and cluster 3 are notably associated with grade 3 and grade 1, respectively (Figure 3C). The ICGC cohort also show similar results (Figure 3B,C).

Overall, the FACs are adapted to the prognosis of HCC patients and associated with traditional TNM staging and grading criteria, which are robustly consistent across multiple cohorts.

### 3.3 The relationship between FACs and immune landscape

**FIGURE 4:**
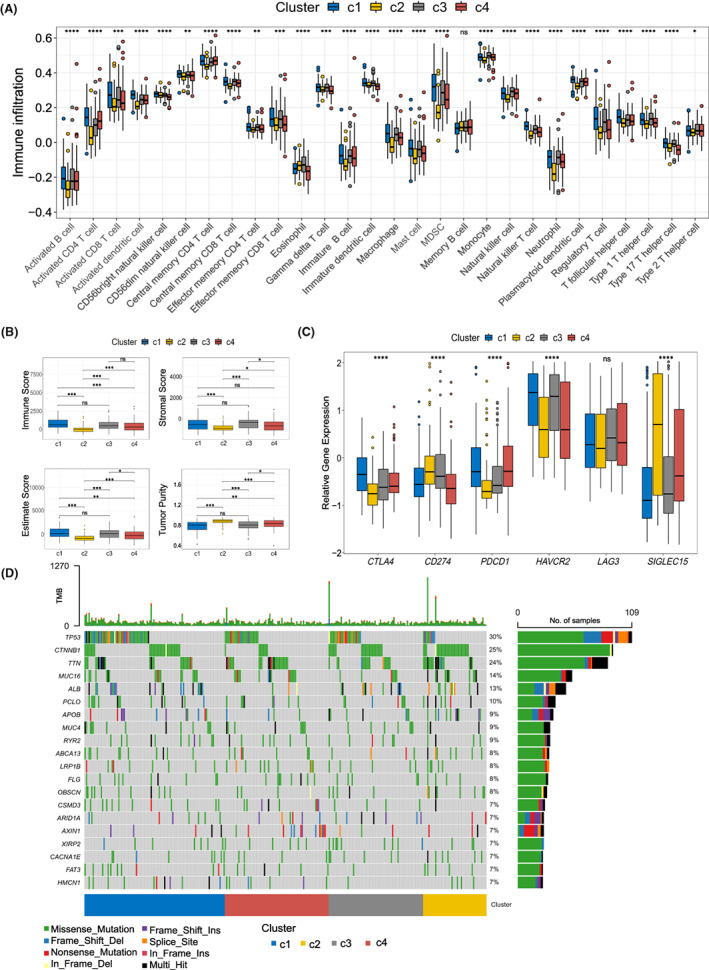
Relationship between FACs and immune landscape. (A) The infiltration status of 28 immune cell types in four clusters. (B) Stromal score, estimate score, immune score and tumor purity of FRPM's clusters. (C) The relative expression levels of immune checkpoint‐associated molecules in four clusters. (D) Twenty highly mutated genes in LIHC ferroptosis subtypes. *p < 0.05, **p < 0.01, ***p < 0.001 and ****p < 0.0001.

It is commonly believed that ferroptosis and relevant immune microenvironment are broadly connected with the development and target therapy of different cancers.^6^, ^18^, ^23^ The scores of most immune cells in cluster 1 was observably higher than those in cluster 2 (Figure 4A). Among the four clusters, a significant reduction in immune and stromal scores, which indicates the inferred fraction of stromal or immune cells in tumor samples,^23^ was found in cluster 2 (Figure 4B). Furthermore, tumors with poorer survival (cluster 1) yielded higher stromal and immune scores than those with good survival (cluster 2) (Figure 4B). Moreover, the expression of six immune checkpoint proteins in the TCGA cohort were analyzed, of which *CTLA4*, *CD274*, *PDCD1*, *HAVCR2*, and *SIGLEC15* were differentially expressed among the FACs in the TCGA cohort (Figure 4C). *CTLA4*, *PDCD1*, and *HAVCR* were significantly upregulated in tumor tissues of cluster 1, while *SIGLEC15* and *CD274* exhibited notably higher expression levels in tumor tissues of cluster 2.

It's well known that higher somatic mutation rates is beneficial to the exploitation of tumor neoantigens.^24^, ^25^ As demonstrated in Figure 4D, the predominantly mutated genes of HCC, including tumor protein P53 (*TP53*), cadherin‐associated protein catenin beta 1 (*CTNNB1*), titin (*TTN*). Moreover, *TP53*, *CTNNB1*, and *TTN*, showed higher frequency in cluster 1 (39%, 48/121), cluster 2 (59.6%, 28/47), and cluster 4 (25.8%, 24/93).

### 3.4 Construction of ferroptosis‐associated prognostic model

**FIGURE 5:**
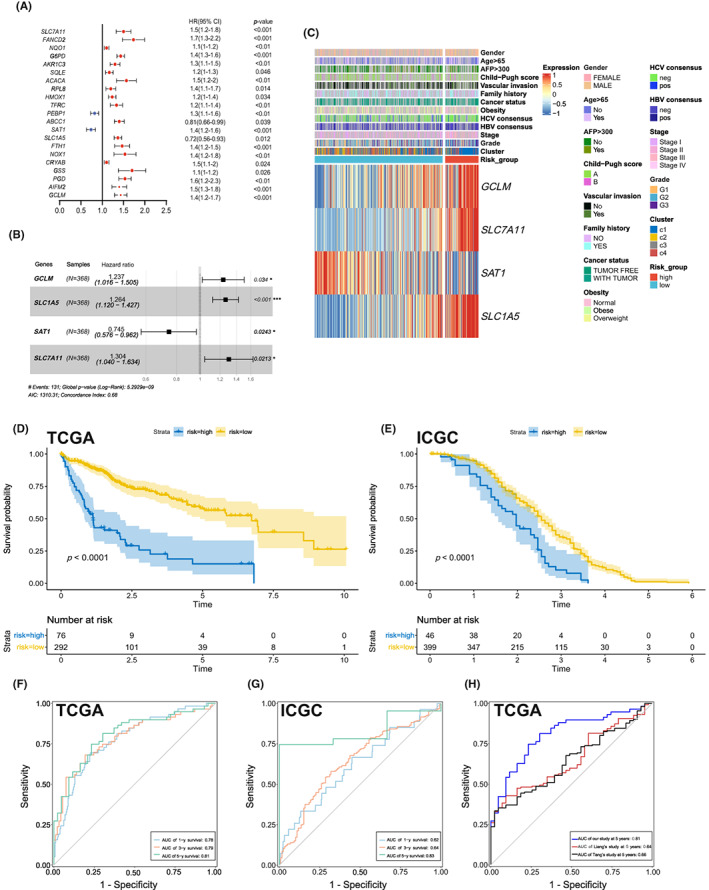
Construction of the ferroptosis phenotype‐related prognostic model (FRPM) for prognostic prediction of HCC. (A) Twenty‐one survival‐related genes were detected by univariate Cox regression analysis. (B) Multivariate Cox proportional hazards regression analysis shows the hazard ratios (HRs) of 4 FDEs with 95% confidence intervals (CIs). (C) Hierarchical cluster analysis using 4 FDEGs from patients with clinical characteristics of LIHC subclasses. (D) Kaplan–Meier survival curves exhibiting the OS of high‐ and low‐risk LIHC patients according to FRPM (training cohort). (E) Kaplan–Meier survival curve of ICGC cohort (validation cohort). (F) ROC curve based on the constructed signature for 1, 3, and 5 years in TCGA cohort. (G) ROC curve based on the constructed signature for 1, 3, and 5 years ‐in ICGC cohort. (H) ROC curves based on our model (blue), Tang's model (red), and Liang's model (black) for 5 years in LIHC‐TCGA cohort, respectively.

To further exploit the relationship between FRGs and the prognosis of HCC patients, 45 FDEGs were extracted and analyzed by univariate Cox regression analysis. The result has shown that the expression of *SLC7A11*, *FANCD2*, *NQO1*, *G6PD*, *AKR1C3*, *SQLE*, *ACACA*, *AIFM2*, *RPL8*, *HMOX1*, *TFRC*, *ABCC1*, *SLC1A5*, *FTH1*, *NOX1*, *CRYAB*, *GSS*, *PGD*, and *GCLM* were defined as the high‐risk markers for OS in LIHC‐TCGA patients. In addition, *PEBP1* and *SAT1* were protective risk markers (Figure 5A).

Different combinations of those genes are evaluated for the quality of statistical models by Akaike information criterion, and the most suitable model is used for constructing multivariate Cox regression models.^17^ Finally, *SLC7A11*, *SLC1A5*, *SAT1*, *GCLM* were identified as independent prognostic factors for overall survival (Figure 5B). Prognostic index = (0.26525 × expression level of *SLC7A11*) + (0.23433 × expression level of *SLC1A5*) + (0.21255 × expression level of *GCLM*) + (−0.29493 × expression level of *SAT1*). High‐risk group (*n* = 76) and low‐risk group (*n* = 292) of HCC patients based on a suitable cutoff value were be distinguished by the formula. The expression patterns of the 4 genes in different clinicopathological groups were exhibited in the heatmap (Figure 5C).

A relatively short OS for high‐risk group in contrasted with low‐risk group was found in Figure 5D (*p* < 0.001). Next, the external HCC cohort (ICGC dataset) also showed the comparably‐similarly result (*p* < 0.001, Figure 5E). To evaluate the prognosis prediction efficiency, time‐dependent ROC curve analysis was applied to HCC patients according to FRPM. As shown in Figure 5F, the Area Under the ROC Curve (AUC) was 0.81 at 5 years, 0.79 at 3 years, and 0.78 at 1 year in the TCGA cohort, respectively. In addition, the AUC values of FRPM were 0.83 (5 years), 0.64 (3 years), and 0.62 (1 years) in the validation ICGC cohort (Figure 5G). Moreover, Our work showed higher prognostic efficiency (AUC = 0.81) compared with previous studies^20^, ^21^ as shown in Figure 5H.

### 3.5 Construction and validation of nomogram

**FIGURE 6:**
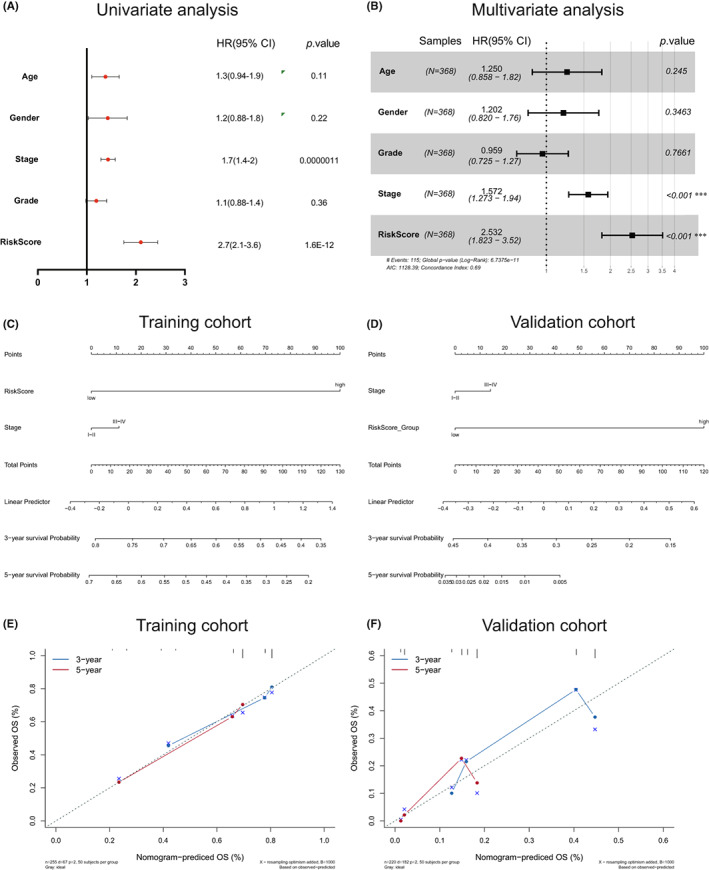
Constructing and validating a nomogram in terms of the FRPM risk score model. (A, B) Relevant clinicopathological items were estimated by uni‐ and multivariate Cox regression analysis. (C, D) The prognostic nomogram through combining the FRPM risk score and clinical‐related prognostic indicators based on the training cohort (C) and validation cohort (D). (E, F) Calibration curves for TCGA (E) and ICGC (F) dataset. The dotted line means a perfect prediction and the blue and red lines indicate the predictive efficiency of the 3 and 5 year OS rates, where the fitness of both color lines to the dotted line describes a good prediction by the model.

To provide a quantitative method containing clinical information to predict the survival probability of HCC patients, a nomogram was constructed based on the FRPM risk score and clinical characteristics related to OS. The univariate analysis showed the FRPM risk score (HR = 2.7, 95% CI 2.1–3.6) and stage (HR = 1.7, 95% CI 1.4–2.0) were positively correlated with the OS of HCC (Figure 6A). Moreover, multivariate Cox analysis indicates that the FRPM risk score (HR = 2.532, 95% CI 1.823–3.52) and stage (HR = 1.572, 95% CI 1.273–1.94) were significantly related to OS (Figure 6B). We integrated the FRPM risk score and stage to establish a nomogram to predicted 3‐ and 5‐year OS in the TCGA training cohort and the ICGC validation cohort (Figure 6C,D). The concordance index (C‐index) indicating the prediction accuracy of the nomogram is 0.711 (95% CI, 0.659–0.762) and 0.612 (95% CI, 0.659–0.762), respectively for the training cohort and validation cohort. A remarkable consensus between actual survival probability and the predicted survival probability for the 3‐ and 5‐ year OS based on the nomogram in both cohorts (Figure 6E,F).

### 3.6 Independent sample validation

**FIGURE 7:**
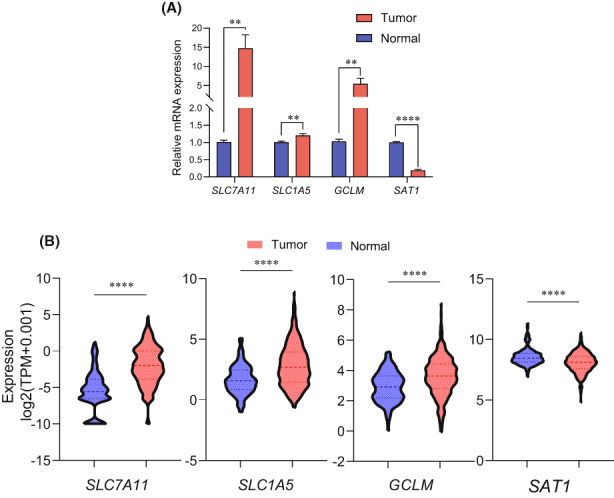
The relevant mRNA expression levels of the four genes between HCC and normal liver tissues. (A) The relevant mRNA expression levels of the four genes in normal (*n* = 6) and HCC tissues (*n* = 6). (B) The four differentially expressed genes in HCC patients (*n* = 375) and non‐tumor liver tissues (*n* = 160) based on the TCGA‐LIHC and GTEx database (160 normal liver tissues). The dotted lines showed in violin plot are upper quartile, median, and lower quartile of samples.

The RT‐PCR results demonstrated that mRNA expression levels of *SLC7A11*, *SLC1A5*, and *GCLM* were notably up‐regulated and *SAT1* was down‐regulated in HCC patients' tumor tissues compared with their ANLTs (Figure 7A), which were consistent with the results from TCGA database analysis (Figure 7B).

## 4 DISCUSSION

In clinical practice, hepatectomy, chemotherapy, and liver transplantation are still the mainstay of HCC treatment.^26^ The development of systemic therapies such as tyrosine kinase inhibitors, immune checkpoint inhibitors, and monoclonal antibodies has challenged the traditional treatment.^27^, ^28^, ^29^ However, the newly numerous immune‐based therapies, such as immune checkpoint inhibitors (anti‐PD1/PD‐L1 and anti‐CTLA4), did not result in prolonged OS and PFS.^30^, ^31^ Therefore, it's necessary to explore the tumor microenvironment in liver cancer. Ferroptosis is defined as an iron‐dependent regulatory cell death procedure, which programmed death mediated by the presence of redox‐active iron, unrepaired phospholipids, and overwhelming peroxidation.^32^, ^33^, ^34^ Wang et.al reported that IFN‐γ‐activated CD8^+^ T lymph cells promoted irreparable lipid peroxidation, and sensitized tumors through mediated the occurrence of ferroptosis.^35^ In addition, ferroptosis can be induced by radiation and pharmacologically augmented by ferroptosis agonists.^36^ Thus, combining ferroptosis with immunotherapy would be a potential appreciated therapeutic approach.

The work herein we have done was to reveal a potential FACs and prognostic indicator of in LIHC‐TCGA cohort. Based on the results of uni‐ and multi‐variate Cox analyses on FDEGs, a newfangled FRPM containing 4 DEGs (*SLC7A11*, *SLC1A5*, *SAT1*, and *GCLM*) was constructed. The results adequately indicate the crucial role of the establishment of ferroptosis relevant prognostic model in HCC and further differentiate different outcomes in patients. Finally, we detected the mRNA expression level of the four candidate genes between HCC tissues and adjacent non‐tumor liver tissues (Figure 6). To entirely exploit the potential of the FRPM, we constructed a nomogram combining the stage, grade age and gender. The calibration curves of 3 and 5 years based on the TCGA and ICGC cohorts' nomograms revealed that the nomogram has excellent predictive efficiency in HCC patients. Therefore, our 4 genes FRPM risk signature can predict the OS of HCC patients and accelerate the selection of optimal treatment approaches.

Interestingly, FACs can reflect the immune landscape of HCC (Figure 4A–D). Cluster 1 showed meaningfully increased scores of activated CD8 T cell, activated B cell, effector memory CD4 T cell and monocyte compared to Cluster 2. High stromal fraction and the expression of *CTLA4*, *PDCD1*, and *HAVCR2* also declare that cluster 1 HCC patients may have a better response to specific immunotherapy. Furthermore, cluster 2 exhibited the lowest CD8^+^ T cell and stromal fraction, indicating an immunologically ‘cold’ phenotype. *TP53* and *CTNNB1* mutations account for a large proportion in cluster 1 (39%, 48/121) and cluster 2 (59.6%, 28/47), respectively (Figure 4D). The abnormally expressed TP53 regulates ferroptosis through regulating the expression of different downstream target molecules.^37^, ^38^ Overexpression of Frizzled‐7, a transmembrane domain receptor for Wnt signaling, alters GPX4‐depended glutathione metabolism and is hyper‐sensitive to ferroptosis.^39^ Therefore, FACs can moderate reflect the immune infiltration and gene mutation of patients, and further guide the clinical treatment of HCC.

SLC7A11, the subunit of membrane transport channel of cysteine and L‐glutamine, distinctly sustains the production of GSH.^40^ The synthesis of GSH, a master endogenous anti‐oxidant, relies on the availability of cystine.^41^ Once the function of SLC7A11 was dampened, while treated with its small molecule inhibitors (erastin) or drug (sorafenib), cystine uptake was reduced and GSH levels could not be maintained, thereby affecting the inhibition of GPX4 on ferroptosis.^42^ In cystine‐deficient melanoma with ferroptosis induced by erastin, ectopic expression of miR‐137 reinforced anti‐tumor activity by suppressing SLC1A5.^43^ TP53, a tumor suppressor, regulates lipid peroxidation through transcriptional induction of spermidine/spermine N1‐ acetyltransferase 1 (SAT1), so as to facilitate the induction of ferroptosis in human osteosarcoma cells.^44^ BTB domain and CNC homolog 1 promote ferroptosis by repressing the transcription of *GCLM*, which driving the synthesis of the antioxidant GSH.^45^, ^46^ It's previously reported that SLC1A5, SLC7A11, and GCLM are negative regulators that restrain ferroptosis in some kinds of cancers, while the remaining SAT1 facilitates ferroptosis.^43^, ^46^, ^47^, ^48^, ^49^, ^50^, ^51^ However, the related mechanism of GCLM and SAT1 in HCC remains to be further elucidated. Together, *SLC7A11*, *SLC1A5*, *SAT1*, and *GCLM* are underlying biomarkers to exhibit the invasiveness‐related molecular characteristics of ferroptosis, which benefit personalized prognostic assessment and effectively combination with different treatments.

Nevertheless, our study had some limitations. First, some prognosis‐related clinicopathological factors were not obtainable in databases, which may potentially influence the prognostic accuracy in the nomogram. Second, although our prognostic model was validated in online database, further validation based on clinical HCC patients is still needed to estimate the performance of the model. Finally, more studies, such as molecular mechanism, prospective clinical utility, and the relationship between risk score and immune activity, are required to further elucidate.

## 5 CONCLUSION

In summary, a newly FRPM of 4 ferroptosis‐associated genes was established in herein study. Our model is independently verified in both LIHC‐TCGA and ICGC databases, offering a candidate model for the prediction of HCC patients' survival. Moreover, this study provides new insight and strategy to effectively stratify patients for substantially predictive, preventive, and personalized management of different subtypes of HCC patients.

## AUTHOR CONTRIBUTIONS

Conceptualization: KF and SX. Data curation and Investigation: SL, YC, YW, YY. Supervision: KF.

## FUNDING INFORMATION

This work was supported by grants 31,900,561, 32,170,726, 32,100,580 and 81,702,949 from National Natural Science Foundation of China, 2019RS1010 and 2021JJ20094 from Hunan Provincial Science and Technology Department (China), 2020CX016 from Central South University.

## CONFLICT OF INTEREST

The authors have declared that no competing interests exist.

## ETHICS STATEMENT

Ethical approval was given by the ethics committee of Xiangya Hospital of Central South University (China) and The First Affiliated Hospital of University of South China. This study is conducted under the principles of the Declaration of Helsinki and the Good Clinical Practice guidelines. A waiver of written informed consents from patients was granted by the ethics Committee of the ethics committee of The First Affiliated Hospital of University of South China.

## Supporting information


Appendix S1

## Data Availability

This study was based on publicly available datasets, including LIHC‐TCGA (https://xenabrowser.net/datapages/), LIRI‐JP‐ICGC (https://dcc.icgc.org/releases/release_28/Projects/LIRI‐JP), and ESTIMATE: (https://bioinformatics.mdanderson.org/estimate/).
