# Supplementary material for: Identification of Ferroptosis‐related molecular model and immune subtypes of hepatocellular carcinoma for individual therapy
Source: Cancer Med. 2022 Jul 16;12(2):2134–47. doi: 10.1002/cam4.5032 (PMC9883587; doi:10.1002/cam4.5032)
Supplement: Supplementary file 1 — Appendix S1 [file CAM4-12-2134-s001.docx]

Supplementary Information

**SUPPLEMENTARY TABLE**

TABLE S1 The metadata of the surgical tissues from human hepatocellular carcinoma (HCC) patients

| Gender | Age | Admission number | Admission time | Dignosis |
| --- | --- | --- | --- | --- |
| Male | 51 | 1389379 | 2018/3/27 16:45 | HCC |
| Male | 70 | 1193011 | 2018/4/25 16:28 | HCC |
| Male | 33 | 1482717 | 2019/5/24 18:19 | HCC |
| Male | 31 | 1504847 | 2019/8/15 10:33 | HCC |
| Male | 71 | 1381566 | 2019/10/31 10:34 | HCC |
| Male | 66 | 1252073 | 2020/5/22 18:12 | HCC |

TABLE S2 The primer sequences used in this paper.

| **The oligonucleotides information** | | |
| --- | --- | --- |
| SLC7A11-homo-Forwad | CGTCCTTTCAAGGTGCCACT | RT-PCR |
| SLC7A11-homo-Reverse | ATCTTCTTCTGGTACAACTTCCAGT | RT-PCR |
| GCLM-homo-Forwad | AGACGGGGAACCTGCTGAA | RT-PCR |
| GCLM-homo-Reverse | CATCTGGAAACTCCCTGACCA | RT-PCR |
| SAT1-homo-Forwad | GTTGCAGAAGTGCCGAAAGA | RT-PCR |
| SAT1-homo-Reverse | TGATCCTATGCCAAAGCCTCTA | RT-PCR |
| SLC1A5-homo-Forwad | TGGGCGCTGCTCTTTTTC | RT-PCR |
| SLC1A5-homo-Reverse | TTCATAGGTGGTAGAGTATGAGCG | RT-PCR |
| ACTB-homo-Forwad | GATTCCTATGTGGGCGACGA | RT-PCR |
| ACTB-homo-Reverse | AGGTCTCAAACATGATCTGGGT | RT-PCR |

**SUPPLEMENTARY FIGURE**


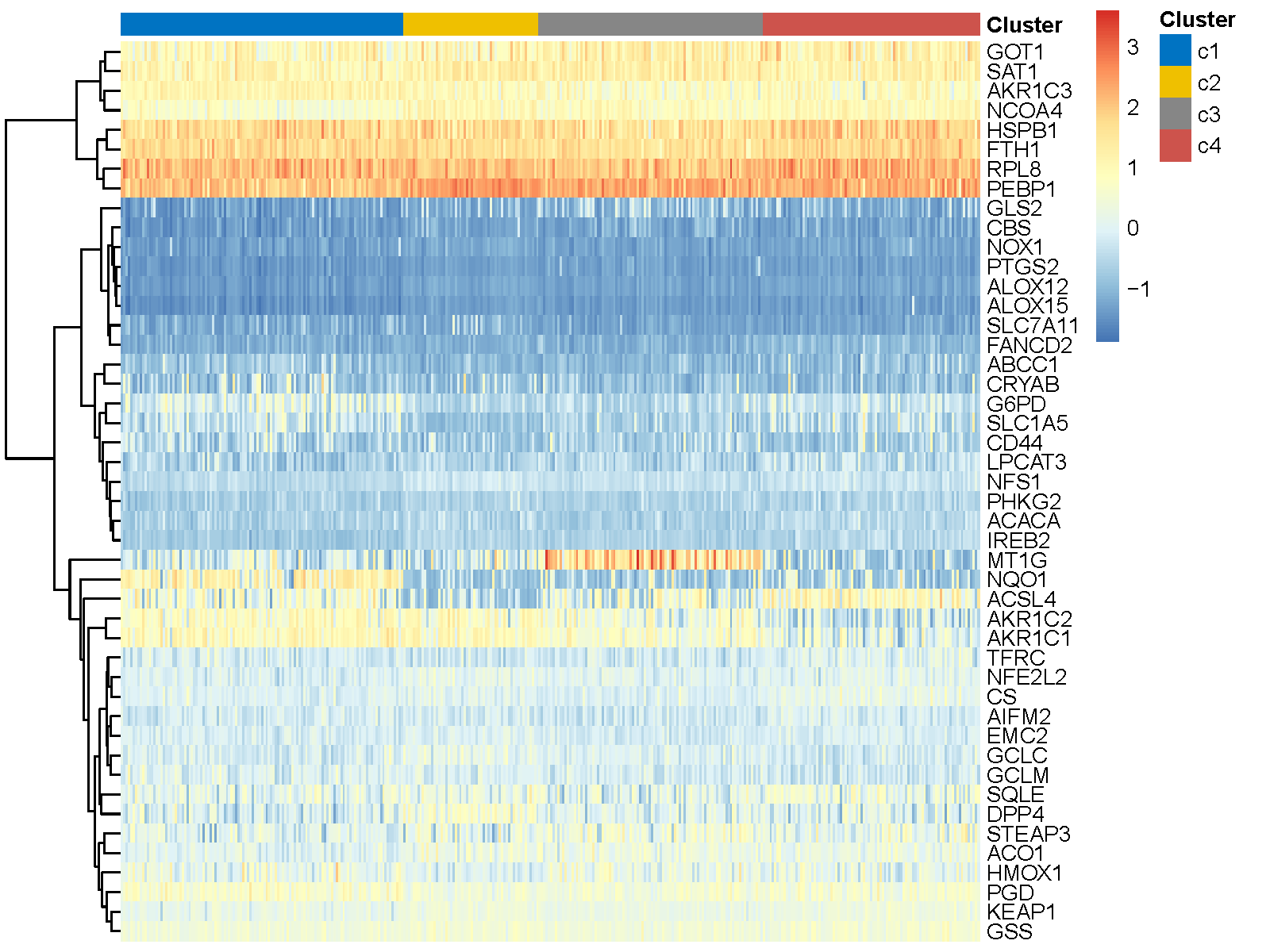


**SUPPLEMENTARY FIGURE LEGEND**

**FIGURE S1 The transcriptomic characteristics of these four categories of LIHC patients.**
